# Supplementary material for: Gastrectomy for Cancer: A 15-Year Analysis of Real-World Data from the University of Athens
Source: Medicina (Kaunas). 2022 Dec 5;58(12):1792. doi: 10.3390/medicina58121792 (PMC9787625; doi:10.3390/medicina58121792)
Supplement: Supplementary file 1 [file medicina-58-01792-s001.zip › Supplemental Table S1.docx]

**Supplemental Table S1.** Patient clinicopathological features and outcomes stratified by tumor location

| **Variable** | **Siewert II**  (N=28; 13.7%) | **Siewert III**  (N=27; 13.1%) | **Gastric Ca**  (N=150; 73.2%) | **Total**  (N=205) | **p-value** |
| --- | --- | --- | --- | --- | --- |
| **Age (y),**  *Mean ± SD* | 67.6 ± 13.4 | 67.8 ± 11.1 | 65.7 ± 13.4 | 66.2 ± 13.1 | 0.51 |
| **Sex** |  |  |  |  | 0.42 |
| *Male* | 15 (46.4%) | 22 (18.5%) | 100 (33.3%) | 137 (66.8%) |  |
| *Female* | 13 (53.6%) | 5 (81.5%) | 50 (66.7%) | 68 (33.2%) |  |
| **Operation** |  |  |  |  | **<0.001** |
| *Total gastrectomy* | 25 (89.3%) | 25 (92.6%) | 47 (31.3%) | 94 (45.8%) |  |
| *Subtotal gastrectomy* | 0 (0%) | 0 (0%) | 101 (67.3%) | 104 (50.7%) |  |
| *Wedge resection* | 0 (0%) | 0 (0%) | 1 (0.7%) | 1 (0.5%) |  |
| *Central (proximal) gastrectomy* | 3 (10.7%) | 2 (7.4%) | 1 (0.7%) | 6 (3%) |  |
| **Morbidity** |  |  |  |  | 0.15 |
| *Yes* | 6 (23.1%) | 11 (44.0%) | 32 (25.8%) | 49 (28.0%) |  |
| *No* | 20 (76.9%) | 14 (56.0%) | 92 (74.2%) | 126 (72.0%) |  |
| **Resection** |  |  |  |  | **0.02** |
| *R0* | 24 (85.7%) | 19 (70.3%) | 140 (93.3%) | 183 (89.3%) |  |
| *R1* | 4 (14.3%) | 7 (25.9%) | 10 (6.7%) | 21 (10.2%) |  |
| *R2* | 0 (0%) | 1 (3.7%) | 0 (0%) | 1 (0.5%) |  |
| **Lymphadenectomy** |  |  |  |  | **0.01** |
| *D0 lymphadenectomy* | 0 (0%) | 0 (0%) | 0 (0%) | 0 (0%) |  |
| *D1 lymphadenectomy* | 18 (64.2%) | 17 (62.9%) | 126 (84.5%) | 161 (78.5%) |  |
| *D2 lymphadenectomy* | 10 (35.8%) | 9 (33.3%) | 21 (14.1%) | 40 (20.0%) |  |
| *D3 lymphadenectomy* | 0 (0%) | 1 (3.8%) | 2 (1.4%) | 3 (1.5%) |  |
| **Lymph nodes dissected,**  *Mean ± SD* | 29.4 ± 13.4 | 33.7 ± 15.9 | 28.5 ± 17.4 | 29.3 ± 16.7 | 0.23 |
| **Positive lymph nodes,**  *Mean ± SD* | 7.5 ± 10.4 | 10.9 ± 11.2 | 8.1 ± 12.6 | 8.4 ± 12.1 | 0.39 |
| **Lauren classification** |  |  |  |  | 0.34 |
| *Diffuse* | 7 (35.0%) | 6 (42.8%) | 54 (42.8%) | 67 (41.9%) |  |
| *Enteric* | 13 (65.0%) | 6 (42.8%) | 56 (44.4%) | 75 (46.9%) |  |
| *Mixed* | 0 (0%) | 2 (14.4%) | 16 (12.8%) | 18 (11.2%) |  |
| **Grade** |  |  |  |  | 0.61 |
| *Grade 1* | 2 (8.0%) | 0 (0%) | 9 (6.7%) | 11 (6.0%) |  |
| *Grade 2* | 9 (36.0%) | 9 (37.5%) | 39 (28.9%) | 57 (30.9%) |  |
| *Grade 3* | 14 (56.0%) | 15 (62.5%) | 87 (64.4%) | 116 (63.1%) |  |
| **Recurrence** |  |  |  |  | 0.25 |
| *Yes* | 5 (35.8%) | 9 (47.4%) | 46 (47.9%) | 60 (46.5%) |  |
| *Censored* | 9 (64.2%) | 10 (52.6%) | 50 (52.1%) | 69 (53.5%) |  |
| **All-cause mortality** |  |  |  |  | 0.17 |
| *Yes* | 9 (56.2%) | 14 (63.6%) | 61 (56.4%) | 84 (57.5%) |  |
| *Censored* | 7 (43.8%) | 8 (36.4%) | 47 (43.6%) | 62 (42.5%) |  |
