# Supplementary material for: Gastrectomy for Cancer: A 15-Year Analysis of Real-World Data from the University of Athens
Source: Medicina (Kaunas). 2022 Dec 5;58(12):1792. doi: 10.3390/medicina58121792 (PMC9787625; doi:10.3390/medicina58121792)
Supplement: Supplementary file 1 [file medicina-58-01792-s001.zip › Supplemental Table S2 (Revised).docx]

**Supplemental Table S2.** Multivariate linear regression model for positive lymph node rates using stepwise selection

| **Variable** | **Beta coefficient** | **Standard error** | **95% Confidence interval** | **p-value** | **R^2^** | **Root mean square deviation** |
| --- | --- | --- | --- | --- | --- | --- |
| **Stage** | 2.6 | 0.34 | 1.92-3.30 | <0.001 | 0.53 | 8.38 |
| **Number of resected lymph nodes** | 0.33 | 0.43 | 0.24-0.41 | <0.001 |  |  |
| *Intercept* | -15.9 | 2.12 | -20.1 to 11.7 | <0.001 |  |  |
