# Supplementary material for: Gastrectomy for Cancer: A 15-Year Analysis of Real-World Data from the University of Athens
Source: Medicina (Kaunas). 2022 Dec 5;58(12):1792. doi: 10.3390/medicina58121792 (PMC9787625; doi:10.3390/medicina58121792)
Supplement: Supplementary file 1 [file medicina-58-01792-s001.zip › Supplemental Table S3 (Revised).docx]

**Supplemental Table S3.** Multivariate logistic regression model for postoperative morbidity using stepwise selection

| **Variable** | **Odds ratio** | **Odds ratio**  *SD* | **Odds ratio**  *95% CI* | **Beta coefficient** | **Beta coefficient**  *SD* | **Beta coefficient** *95% CI* | **p-value** | **Pseudo R^2^** |
| --- | --- | --- | --- | --- | --- | --- | --- | --- |
| **Adjuvant chemotherapy** | 0.35 | 0.16 | 0.14-0.88 | -1.02 | 0.46 | -1.92 to -0.12 | 0.03 | 0.03 |
| *Intercept* | 0.67 | 0.24 | 0.32-1.38 | -.04 | 0.37 | -1.13 to 0.32 | 0.27 |  |

SD: standard deviation; 95% CI: 95% Confidence interval
