# Supplementary material for: Gastrectomy for Cancer: A 15-Year Analysis of Real-World Data from the University of Athens
Source: Medicina (Kaunas). 2022 Dec 5;58(12):1792. doi: 10.3390/medicina58121792 (PMC9787625; doi:10.3390/medicina58121792)
Supplement: Supplementary file 1 [file medicina-58-01792-s001.zip › Supplemental Table S4.docx]

**Supplemental Table S4**. TNM staging stratified by tumor location

| **Variable** | **Siewert II**  (N=28; 13.7%) | **Siewert III**  (N=27; 13.1%) | **Gastric Ca**  (N=150; 73.2%) | **Total**  (N=205) | **p-value** |
| --- | --- | --- | --- | --- | --- |
| **Tumor (T)** |  |  |  |  | 0.14 |
| *TIS* | 0 (0%) | 0 (0%) | 1 (0.6%) | 1 (0.5%) |  |
| *T1A* | 0 (0%) | 1 (3.8%) | 10 (6.7%) | 11 (5.3%) |  |
| *T1B* | 3 (10.8%) | 0 (0%) | 18 (12.0%) | 21 (10.2%) |  |
| *T2* | 2 (7.1%) | 5 (18.5%) | 12 (8.0%) | 19 (9.2%) |  |
| *T3* | 16 (57.1%) | 9 (33.3%) | 59 (39.3%) | 84 (40.9%) |  |
| *T4A* | 7 (25.0%) | 9 (33.3%) | 46 (30.7%) | 62 (30.3%) |  |
| *T4B* | 0 (0%) | 3 (11.1%) | 4 (2.7%) | 7 (3.5%) |  |
| **Lymph nodes (N)** |  |  |  |  | 0.12 |
| *N0* | 7 (25.0%) | 2 (7.4%) | 44 (29.6%) | 53 (25.9%) |  |
| *N1* | 4 (14.3%) | 4 (14.8%) | 20 (13.4%) | 28 (13.8%) |  |
| *N2* | 10 (35.7%) | 6 (22.2%) | 32 (21.4%) | 48 (23.6%) |  |
| *N3* | 7 (25.0%) | 15 (55.6%) | 53 (35.6%) | 75 (36.7%) |  |
| **Metastasis (M)** |  |  |  |  | 0.33 |
| *M0* | 27 (96.4%) | 27 (100%) | 140 (93.3%) | 194 (94.6%) |  |
| *M1* | 1 (3.6%) | 0 (0%) | 10 (6.7%) | 11 (5.4%) |  |
| **Stage** |  |  |  |  | 0.61 |
| *0* | 0 (0%) | 0 (0%) | 1 (0.6%) | 1 (0.5%) |  |
| *IA* | 3 (10.8%) | 1 (3.7%) | 15 (10.1%) | 19 (9.3%) |  |
| *IB* | 1 (3.5%) | 1 (3.7%) | 15 (10.1%) | 17 (8.3%) |  |
| *IIA* | 2 (7.1%) | 1 (3.7%) | 18 (12.1%) | 21 (10.3%) |  |
| *IIB* | 4 (14.2%) | 4 (14.9%) | 19 (12.7%) | 27 (13.2%) |  |
| *IIIA* | 2 (7.1%) | 3 (11.1%) | 15 (10.1%) | 20 (9.9%) |  |
| *IIIB* | 11 (39.3%) | 11 (40.7%) | 32 (21.4%) | 54 (26.5%) |  |
| *IIIC* | 3 (10.8%) | 6 (22.2%) | 24 (16.1%) | 33 (16.2%) |  |
| *IV* | 2 (7.1%) | 0 (0%) | 10 (6.8%) | 12 (5.8%) |  |
