# Supplementary figures and images for: Gastrectomy for Cancer: A 15-Year Analysis of Real-World Data from the University of Athens
Source: Medicina (Kaunas). 2022 Dec 5;58(12):1792. doi: 10.3390/medicina58121792 (PMC9787625; doi:10.3390/medicina58121792)

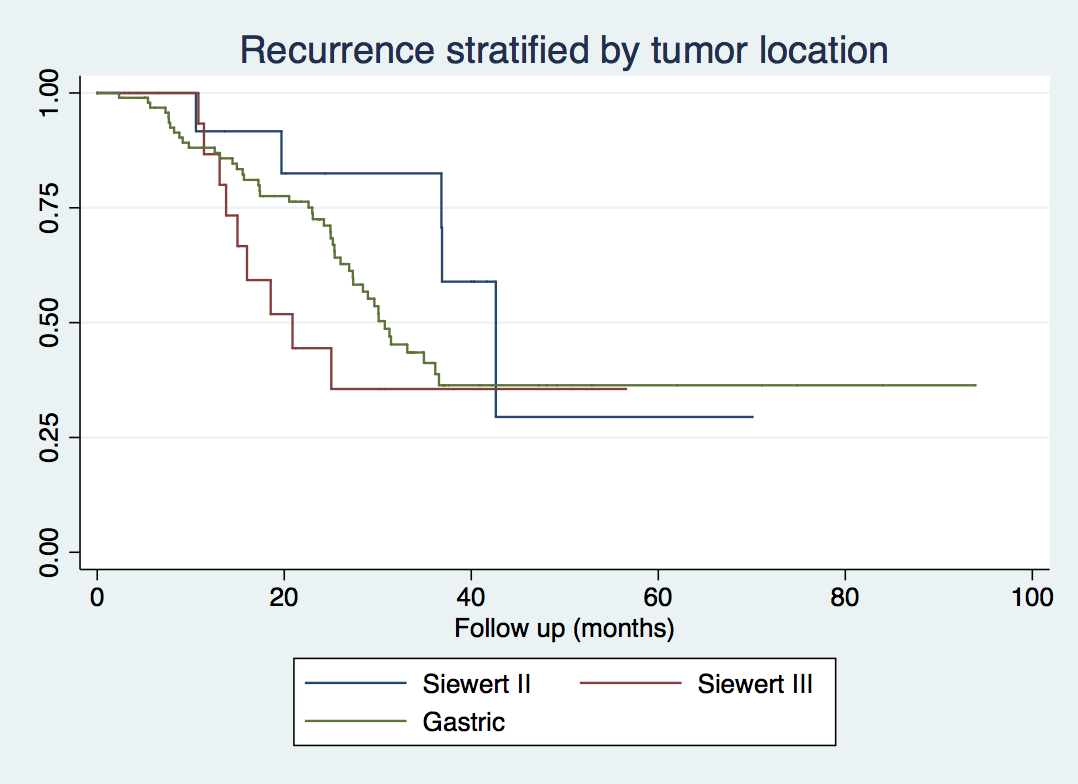

Supplement: Supplementary file 1 [file medicina-58-01792-s001.zip › Supplemental Figure S1. Recurrence by location.tif]

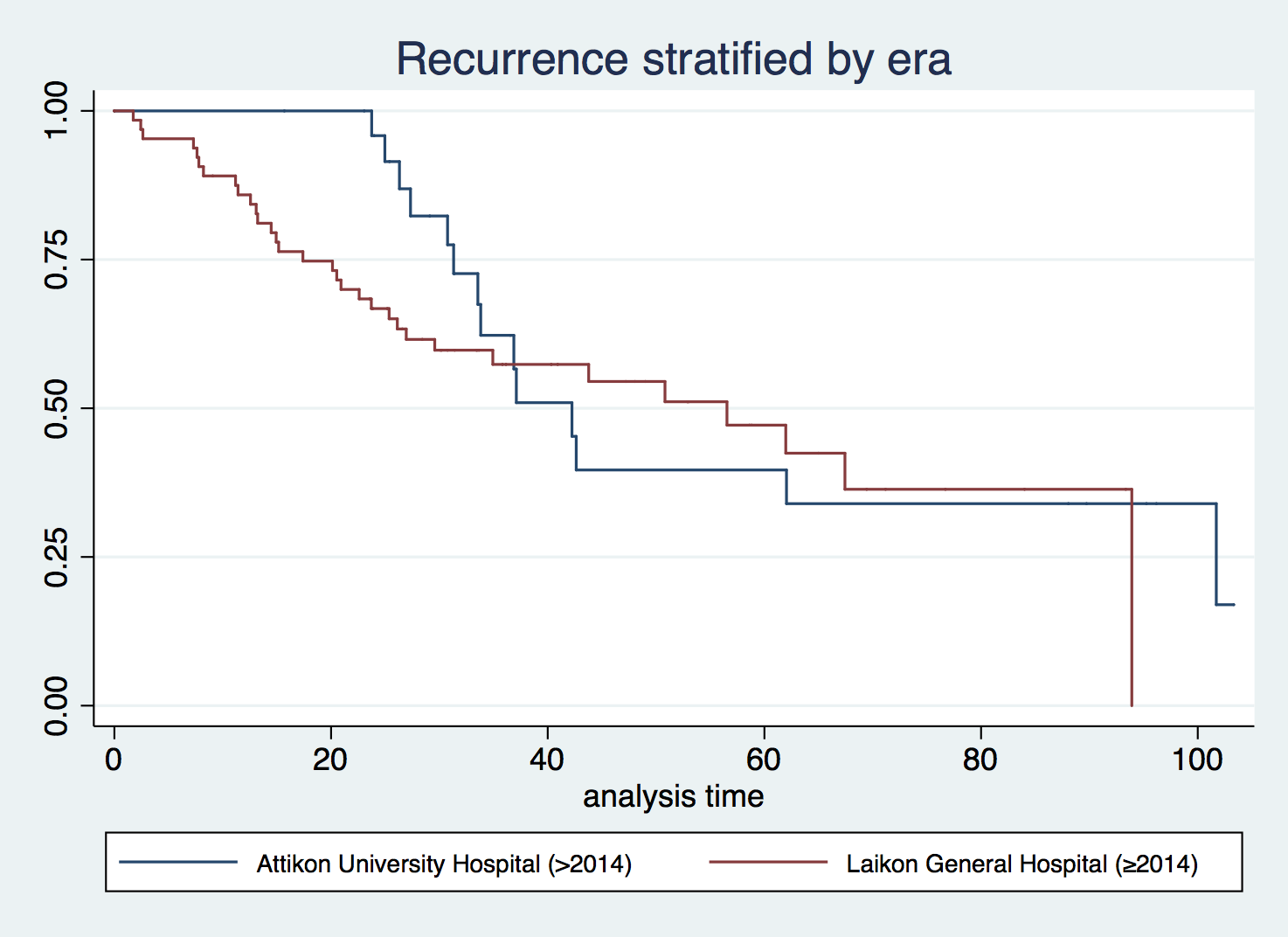

Supplement: Supplementary file 1 [file medicina-58-01792-s001.zip › Supplemental Figure S2. Recurrence by era.tif]

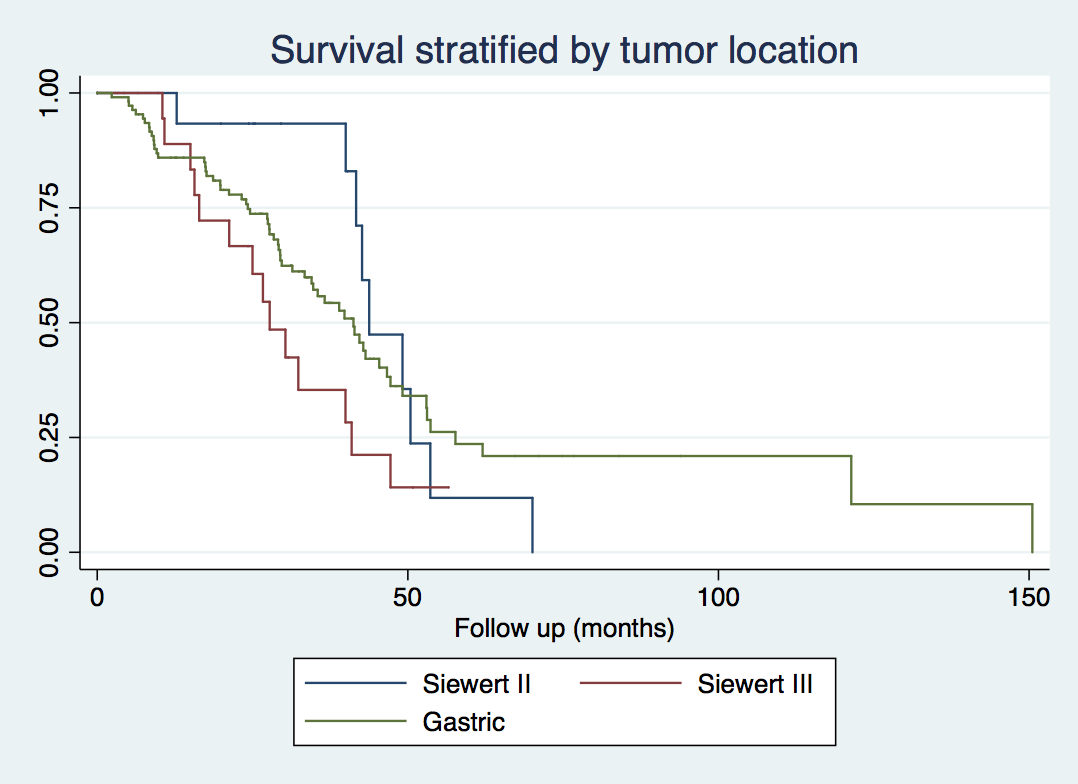

Supplement: Supplementary file 1 [file medicina-58-01792-s001.zip › Supplemental Figure S3. Survival by location.tif]

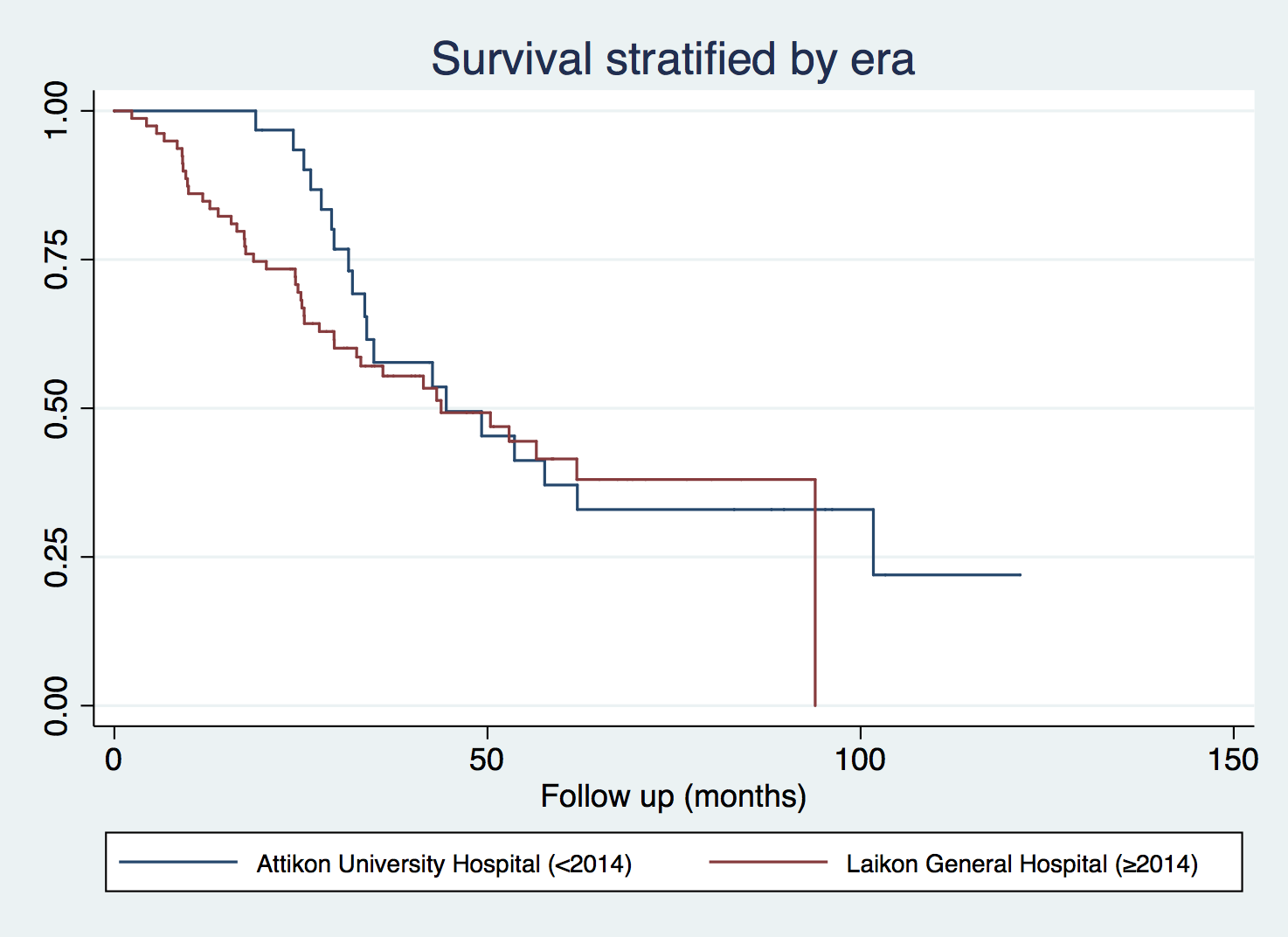

Supplement: Supplementary file 1 [file medicina-58-01792-s001.zip › Supplemental Figure S4. Survival by era.tif]
